# Shifting attention between modalities: Revisiting the modality-shift effect in autism

**DOI:** 10.3758/s13414-021-02302-4

**Published:** 2021-04-30

**Authors:** Daniel Poole, Eleanor Miles, Emma Gowen, Ellen Poliakoff

**Affiliations:** 1 Division of Neuroscience and Experimental Psychology, University of Manchester, Oxford Road, Manchester, M139PL UK; 2 School of Psychological Sciences, University of Sussex, Brighton, Sussex, UK

**Keywords:** Selective Attention, Autism drift, Diffusion model, Multisensory processing

## Abstract

Selective attention to a sensory modality has been observed experimentally in studies of the modality-shift effect – a relative performance benefit for targets preceded by a target in the same modality, compared to a different modality. Differences in selective attention are commonly observed in autism and we investigated whether exogenous (automatic) shift costs between modalities are increased. Autistic adults and neurotypical controls made speeded discrimination responses to simple visual, tactile and auditory targets. Shift costs were observed for each target modality in participant response times and were largest for auditory targets, reflective of fast responses on auditory repeat trials. Critically, shift costs were similar between the groups. However, integrating speed and accuracy data using drift-diffusion modelling revealed that shift costs in drift rates (reflecting the quality of information extracted from the stimulus) were reduced for autistic participants compared with neurotypicals. It may be that, unlike neurotypicals, there is little difference between attention within and between sensory modalities for autistic people. This finding also highlights the benefit of combining reaction time and accuracy data using decision models to better characterise selective attention in autism.

## Introduction

Perceiving and interacting with the world around us involves rapid and effective shifts in our focus of attention. For instance, many people in a given city safely navigate their route through the sensory barrage of the high street whilst avoiding obstacles, checking their phone and drinking hot coffee. Indeed, selective attention can be directed endogenously (voluntarily) towards a particular sensory modality (e.g. vision, touch, hearing) for preferential processing (Posner et al., 1976) in a manner similar to cueing of visual spatial attention (Posner, 1980). When presented with a symbolic cue that informs the participant of the modality of an upcoming target, response times (RTs) are reduced in comparison to trials where the cue is non-informative (Spence & Driver, 1997). Attention can also shift between modalities without explicit instructions; the modality of a cue can exogenously capture attention, even if it does not provide any information or require a response (referred to herein as cue-target paradigms). For instance, when participants are presented with a task-irrelevant cue immediately prior to a target, responses are faster on repeat trials (e.g., visual cue followed by a visual target) relative to shift trials (visual cue followed by an auditory target), a modality-shift effect (MSE; Turatto et al., 2002). MSEs have also been observed using target-target paradigms where all the stimuli presented require a judgement and the MSE reflects the shift in attention from the modality of the preceding trial (Spence et al., 2001). Measurement of event-related potentials to visual-tactile stimuli has revealed increased N1 amplitudes following shift trials relative to repeat trials that were not modulated by the modality of the shift, that is, observed for both visual to tactile and tactile to visual shifts (Tollner et al., 2009). N1 is an early potential that is typically observed when an unexpected stimulus (relative to previous stimuli) is presented. This observation led to the suggestion that the MSE reflects supramodal control processes involved in modality shifting (the modality weighting account).

Attentional control may be affected in a number of populations and the MSE paradigm has been used to investigate attention across the senses, most commonly in studies of schizophrenia (see Mannuzza, 1980, for a review). This work has indicated that MSEs between visual and auditory targets are increased in the condition (Ferstl et al., 1994; Krag et al., 2017; Spring, 1980). The MSE has also been investigated in dyslexia (Harrar et al., 2014). Participants with dyslexia exhibited an increased MSE when shifting from visual to auditory targets in comparison with controls. These findings suggest that the control processes involved in shifting attentional weight between the senses may be less effective in these neurodivergent populations. In the current investigation, we explored the nature of the MSE in autism.

Autism is a neurodevelopmental condition that affects a number of aspects of cognition and behaviour. This can include social interaction, communication, and restricted or repetitive patterns of behaviour (American Psychiatric Association, 2013). Atypical cognitive control has been postulated to underlie many of the symptoms of autism (Hill, 2004; Pennington & Ozonoff, 1996; although see Geurts et al., 2009). In particular, numerous studies have suggested that aspects of attention are divergent in autism, including delayed disengagement of spatial attention (Elsabbagh et al., 2013; Landry et al., 2009), increased interference from distractors (Adams & Jarrold, 2012; Christ et al., 2011), and processing of non-target stimuli under conditions of high perceptual load (Remington et al., 2009; Remington et al., 2012). Everyday environments are rich in multisensory information, and as such a reduced ability to effectively shift attention between the senses could lead to perceptually overwhelming experiences.

Indeed, questionnaire studies have shown that differences in reactivity to everyday sensory stimuli are commonly reported in autism (Baranek et al., 2006; Lane et al., 2011; Leekam et al., 2007), including both hyper- and hypo- reactivity to everyday stimuli. However, only a limited number of studies have investigated crossmodal shifting in autism to date, and the methodology, findings and interpretations of the existing work has been mixed (see Table 1 for summary), in common with the wider literature on attention in the condition (Ames & Fletcher-Watson, 2010).

**Table 1:** Studies investigating the modality-shift effect (MSE) in autism

Study	Participants	Task judgement	Task type	Difference in shift costs?	Comments
Charbonneau et al. (2019)	14 autistic adults and 14 age-, gender- and handedness-matched NT controls	Detection	Exogenous (Target-Target)	No	• Visual-tactile task • Included both unimodal and visuotactile targets
Courchesne et al. (1994)	8 autistic children and three control groups: 6 cerebellar patients, 8 NT matched on age and 8 matched on IQ	Detection	Exogenous (Cue-Target)	Increased in autism	• Spatial confound • Participant required to remember rule change following oddball stimuli.
Haigh et al. (2016)	15 autistic adults and 15 age- and gender-matched NT controls	Discrimination	Endogenous (symbolic cue)	No	• Spatial confound • Stimuli titrated to threshold • All stimuli audiovisual • Included responses cues. The use of invalid response cues may have encouraged dividing attention rather than shifting
Occelli et al. (2013)	14 autistic children and 17 age-matched NT controls, no IQ matching	Detection	Exogenous (Cue-Target)	Reduced in autism	• Modality of target was predictable as blocked, cue alternated trial to trial
Murphy et al. (2014)	20 autistic children and 20 age-, IQ- and gender-matched NT controls	Discrimination	Endogenous (symbolic cue)	No	• Stimuli titrated to threshold • Included both unimodal and audiovisual targets
Williams et al. (2013)	33 autistic children,42 autistic adults and age-, IQ- and gender-matched NT groups of 33 children and 42 adults	Detection	Exogenous (Target-Target)	Increased in autistic children	• Included within modality shifts

*NT* neurotypical

In an early study, adolescents respond to visual and auditory oddball targets among sequences of visual and auditory stimuli (for instance, a red flash amongst a sequence of green flashes), with the oddball stimuli also serving as a cue to shift the focus of attention between the modalities (Courchesne et al., 1994). Participants with autism showed reduced detection of targets when 2.5 s or less had passed since the previous target (and thus shift in attention). A developmental study described divergent performance in crossmodal shifting in different age groups in autism (Williams et al., 2013). Children with autism produced a greater MSE than neurotypicals (NTs) when shifting from auditory targets to visual. Adults with autism produced slower RTs across the conditions, but did not show any differences from adult controls in crossmodal shifting. Two recent studies¸ that used auditory and visual stimuli set to the thresholds of individual participants in cue-target tasks¸ observed no group differences in the MSE in autistic children (Murphy et al., 2014) and autistic adults (Haigh et al., 2016). Autistic adults also produced similar shift costs to controls in a target-target study requiring responses to spatially aligned and misaligned visual, tactile and visuotactile stimuli (Charbonneau et al., 2019).

Finally, one study has described a *reduced* MSE in autism (Occelli et al., 2013). Adolescent participants were presented with audiovisual stimuli in a cue-target detection task. The modality of the target remained constant across blocks, while the cue stimulus varied from trial to trial. When the interval between the cue and target was 1,000 ms, autistic participants produced a smaller shift cost for audition and a negative shift cost (benefit) for vision (meaning that responses were faster following a shift cue in comparison to a repeat cue).

There are a number of methodological considerations in studies of the MSE, many of which have been discussed elsewhere (Miles et al., 2011; Spence et al., 2001; Spence & Driver, 1997), but are of particular importance when working with neurodivergent groups such as with autism. First, Spence and Driver (1997) noted that in many studies of the MSE, crossmodal stimuli were presented from separate spatial locations. This presents an issue when measuring shifts in attention between different sensory modalities, as shifting attention between the different spatial locations will also contribute to the shift costs. This is particularly problematic as attentional orienting and control across space may be less effective in autism (Burack, 1994; Poole et al., 2015; Townsend et al., 1996). This spatial confound applies to both the Courchesne et al. (1994) and Haigh et al. (2016) studies in which visual stimuli were presented on a monitor and auditory stimuli through headphones.

Second is the use of detection paradigms, requiring a rapid response following the presentation of a stimulus (e.g., Courchesne et al., 1994; Occelli et al., 2013; Williams et al., 2013) versus discrimination paradigms, which require speeded discrimination of a dimension of the stimuli (e.g., Haigh et al., 2016; Murphy et al., 2014). Spence et al. (1997) noted that MSEs observed in detection tasks may simply reflect the participant lowering their response criterion in ipsimodal conditions, rather than reflecting attention towards a sensory modality (i.e., preparing to respond quickly to a stimulus in the same modality). In fact, all of the studies that reported differences between autistic and NT participants used detection tasks, meaning that the effect could simply reflect differences in response strategies, rather than less effective attentional control.

Third is the use of cue-target (used in Haigh et al., 2016; Murphy et al., 2014; Occelli et al., 2013) versus target-target (used in Courchesne et al., 1994; Williams et al., 2013) paradigms for investigating the MSE. The use of cues can be problematic, as the extent to which the participant attends to the cue may vary between trials, individuals and groups (see Miles et al., 2011). For instance, in the Occelli et al. (2013) study the modality of the target stimuli was blocked, meaning that an effective strategy would be to direct attention to each target modality and attempt to suppress the cue. Thus, differences in how the groups attended the cue rather than the MSE could explain the observed group differences. A further issue is that the processing of symbolic cues (used in Haigh et al., 2016; Murphy et al., 2014), which can include pictorial or semantic information priming the upcoming modality, could differ between the groups (see Ames & Fletcher-Watson, 2010). It is also worth noting that symbolic cues tend to be presented via a computer screen in the visual modality, which could reintroduce spatial effects and affect the measurement of shifts to non-visual stimuli. Finally, exogenous MSEs are of particular interest in autism as there is evidence from the wider literature that endogenous spatial orienting is comparable to NTs, whereas aspects of exogenous orienting may be affected (Grubb et al., 2013; Landry et al., 2009; Renner et al., 2006).

In summary, there are only a small number of studies investigating MSEs in autism, and these contain heterogenous methods, sample sizes and findings. When taking the confounds described above into account, there is equivocal evidence regarding differences in crossmodal shifting in autism as the majority of previous studies have not investigated the MSE effectively. In the present study, we conducted a comprehensive investigation into MSE in autistic adults by using exogenous crossmodal shifting in autistic and NT adults. In order to address the methodological issues described above, we presented participants with co-located stimuli in a discrimination task for visual, tactile and auditory targets, using a target-target paradigm. The inclusion of shifts between all pairings of visual, auditory and tactile targets is a novel addition, providing insight into tactile attention as well as making it possible to separate shifts in attention *to* and *from* a given modality, which is not possible when investigating two modalities (see Spence et al., 2001). For instance, it would not be possible to determine whether an MSE observed for visual-auditory shifts was a consequence of reduced efficiency in shifting away from vision, or toward audition in a two-modality experiment. If the MSE was also observed for visual-tactile, or tactile-auditory shifts in a three-modality experiment then these explanations could be disentangled.

In line with previous work, we expected to observe a MSE for each sensory modality, with RT on crossmodal trials expected to be increased relative to ipsimodal trials. If the MSE is impacted in autism, we would expect shift costs – the difference in RT between shift and repeat trials – to the visual, tactile and auditory targets to be increased in autistic participants in comparison to controls.

## Method

### Participants

There were 48 participants (24 in the autistic group, 24 in the NT group). Participants in each group were matched for age, full-scale IQ, gender and handedness (see Table 2 for demographic information). Six participants in each group were female. Two participants in each group were left-handed as assessed using the Edinburgh Handedness Inventory (Oldfield, 1971). All participants in the autistic group had previously received a diagnosis from a trained professional, which we confirmed using module 4 of the Autism Diagnostic Observation Schedule-2 (ADOS-2; Lord et al., 2012) by a certified assessor. Participants in the NT group completed the Autism Quotient (AQ; Baron-Cohen et al., 2001), which is a questionnaire measure assessing autistic personality traits in the general population. None of the NT participants produced a total score of 32 or higher, which is the suggested cut-off for clinical interest. Participants in both groups (aside from one autistic participant) completed the Glasgow Sensory Quotient (GSQ; Robertson & Simmons, 2013), which is a questionnaire measure of everyday sensory reactivity. Autistic participants reported both increased hyper- and hypo-sensory reactivity to everyday stimuli.

**Table 2:** Participant characteristics

	Autistic (n = 24)	NT (n = 24)	t (46)	*p*	d
Age,	30.58 ± 7.40	30.17 ± 7.06	0.20	.843	0.06
FSIQ	118.41 ± 10.15	116.88 ± 10.58	0.52	.609	0.15
ADOS	8.83 ± 2.53	-	-	-	-
AQ	-	16.05 ±8.05	-	-	-
GSQ Hyper	38.78 ± 14.68	18.96 ± 8.18	5.75	<.001	1.68
GSQ Hypo	36.74 ± 11.03	18 ± 1.78	6.48	<.001	1.89

Mean ± SD age, full-scale IQ (FSIQ) as measured using the Weschler abbreviated scale of adult intelligence (Weschler, 2008), total score on the Autism Diagnostic Observation Schedule-2 (ADOS), total score on the Autism Quotient (AQ) and score on the hyper- and hypo-responsiveness components of the Glasgow Sensory Quotient (GSQ)

Ethical approval was obtained from the University of Manchester Ethics Committee and informed consent was obtained in writing from all participants.

### Apparatus and stimuli

Participants sat at a desk in a dimly lit room and were asked to focus on a grey cross (10 mm) presented in the centre of the screen throughout the experiment. The participant’s foot depressed two foot pedals, one with their heel and one with their toe. Participants responded to target stimuli by briefly lifting the toe pedal in response to continuous stimuli and the heel pedal in response to pulsed stimuli.

All stimuli were delivered through a Tacamp amplifier (Dancer Design, St Helens, UK) and controlled through E-Prime (version 1.2. Psychology Software Tools Inc, USA). Auditory stimuli (sine wave, 440 Hz, 0.8 AMPs) were delivered through a speaker that was placed on a foam cube 52 cm in front of the participant at the body midline. A bone conductor (Oticon Limited, B/C 2-PIN, 100Ω, Hamilton, UK) was embedded in a 65 x 85 mm foam block that was positioned directly in front of the speaker. The bone conductor was attached to the index finger of the participant’s dominant hand using double-sided adhesive. Vibrations were generated using sound files playing white noise (4,410 Hz, 1 AMPs). A single red LED was embedded in a black plastic cube (25 mm) positioned at the tip of the participant’s index finger. This meant that the stimuli from the three modalities were co-located.

Stimuli in each modality were presented as either pulsed or continuous targets. Tactile targets were either continuous 200-ms vibrations or a series of six pulses lasting 200 ms overall (each pulse was generated by 5-ms bursts of white noise separated by 40 ms of silence). Visual targets were either a continuous 200-ms flash of the red LED or a series of six pulses lasting 200 ms overall (each pulse was generated by the LED shifting on for 1 ms separated by 49 ms off). Auditory targets were either a continuous 200-ms tone (1,000 Hz) or a series of six pulses lasting 200 ms overall (each pulse was generated by 1-ms tones separated by 49 ms of silence). Throughout the experiment, white noise (~75 dB SPL) was played through headphones to block out background sound and to prevent the participant hearing the sounds emitted by the bone conductor. All stimuli were clearly suprathreshold as confirmed by each participant before beginning the practice procedure.

### Procedure

The participant was instructed that the task was to determine whether the stimulus was continuous or pulsed on each trial. They were told that this stimulus could be visual, tactile or auditory, and that they should respond as quickly and accurately as possible. The participant was asked to maintain central fixation throughout every trial.

There were 60 trials in each condition. Participants completed six blocks of 91 trials, with a short break between each block. Trials were presented in a pseudorandomised order so that there were equal numbers of modality repeats and shifts. In addition, response repeats (whether successive trials required the same foot-pedal response) were equal across all conditions. The inter-trial interval (ITI) was 1,000 ms, 1,600 ms or 2,000 ms with an equal number of repeats of each ITI for modality and response shifts/repeats. If the participant took longer than 1,000 ms to respond to a target then they were presented with a warning message for 1,000 ms. All participants received the same six pseudorandomised blocks and the order of these blocks was counterbalanced within each group.

Prior to commencing the experimental task, participants were first familiarised with the stimuli until the experimenter was confident that they could distinguish between continuous and pulsed targets in each modality. To familiarise the participant with the stimuli they were presented with each pulsed then each continuous stimulus while the appropriate response was displayed on-screen. They then completed two practice blocks of 40 trials.

### Data analysis

Median RT to correct responses were calculated. Errors were also calculated and are displayed in Fig. S1 of the Supplementary Materials (available on the study Open Science Framework page, see Results section). One autistic participant who produced a very high error rate in every condition (> 50% error) was removed. As we were interested in the performance to targets in respect of the preceding trial, the first trial in each block was not analysed. Trials in which participants took longer than 1,000 ms to respond (misses, autistic group, 0.49% of trials; NT group, 0.34% of trials) or made an anticipatory response where the RT was < 200 ms (autistic group, 0.60% trials; NT group, 0.34% of trials) were excluded from analysis. Trials following misses were also removed from further analysis as the presentation of the visual warning message would impact on attention shifting in the subsequent trial. A further three autistic and three NT participants were removed as they produced misses on ≥ 25% of trials, meaning that 50% of their data was unusable. This gave a final sample size of 20 autistic participants and 21 neurotypical participants. Outliers in individual responses were identified using the non-recursive outlier removal procedure described by van Selst and Jolicoeur (1994) using the* trimR* package (Grange, 2015; autistic group, 0.02% of trials; NT group, 0.02% of trials).

Median RTs were compared using a 3 × 3 × 2 mixed permutation ANOVA with within participants factors of Current Target Modality (Visual, Tactile, Auditory) and Previous Target Modality (Visual, Tactile, Auditory), and a between-participants factor of Group (Autistic, NT) using the *R permuco* package (Frossard & Renaud, 2018). The Bonferroni correction for multiple comparisons was applied to all follow-up tests; non-parametric effect sizes (Cliff’s Delta) were calculated using the *effSize* package (Torchianio, 2020). Differences in the shift costs between the modalities would be indicated by a significant interaction between current and previous target modalities. To test which modalities showed an MSE, performance in repeat trials were compared with switch in each modality. The critical test of alterations in the MSE in the autistic group was the three-way interaction.

## Results

Data and the analysis code are available on the Open Science Framework (https://osf.io/emqbk/).

**Fig. 1:**
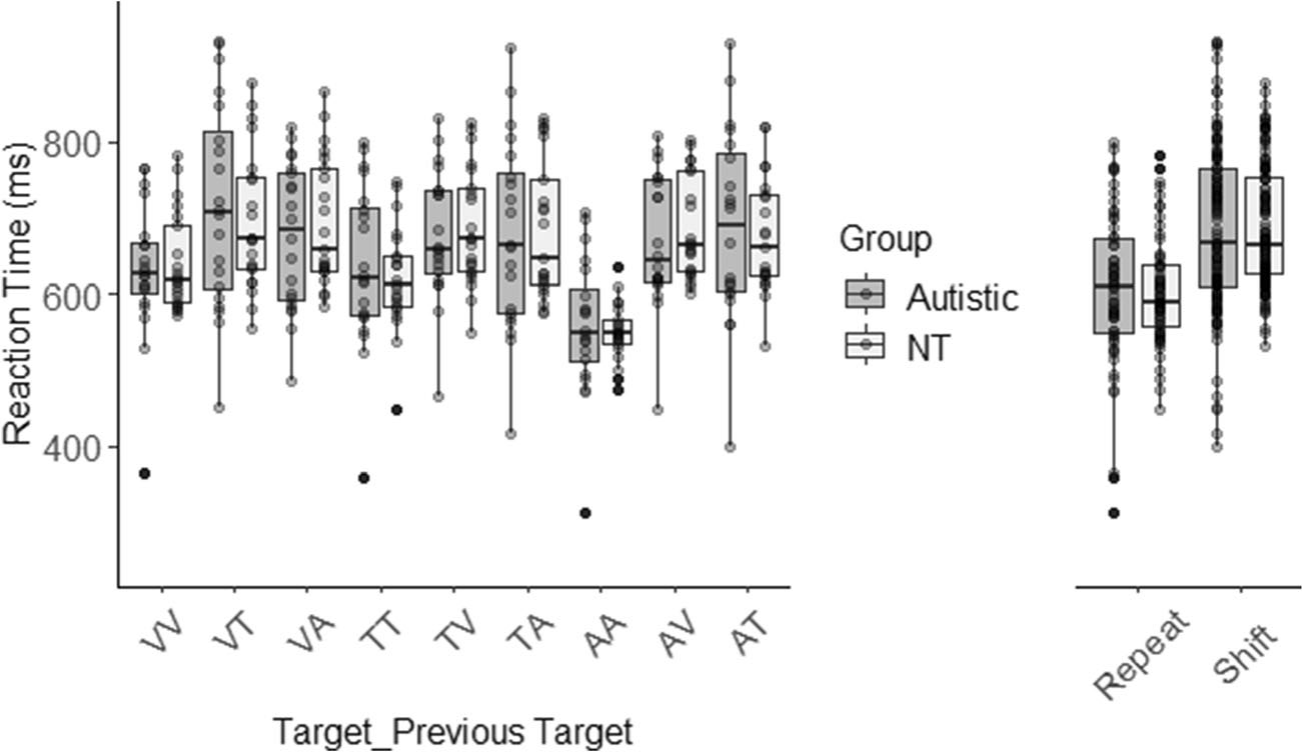
Boxplots displaying reaction time (RT; ms) to each target modality for each previous target modality. The autistic group are shown in grey and the neurotypical (NT) group in white. Data points are individual participant median RT in that condition

RTs to each target are represented in Fig. 1. There was an MSE for each target modality whereby RT on repeat trials were faster than shift trials. RTs were comparable between the groups and, critically, there was no three-way interaction indicating that the MSE was not different between the groups.

This was confirmed using a mixed permutation ANOVA [Current Target Modality (Visual, Tactile, Auditory) x Previous Target Modality (Visual, Tactile, Auditory) x Group (Autistic, NT)], which revealed a significant effect of Current Target Modality (F (2, 351) = 5.34, *p* = .008). Responses to auditory targets were faster than visual (*p* = .004, d = 0.20 95% CI [0.06, 0.34]). The difference between auditory and tactile targets (*p* = .047, d = 0.16 95% CI [0.01,0.29]) and visual and tactile targets (*p* = .326, d = 0.06, 95% CI [-0.09, 0.20]) did not reach statistical significance.

There was also a main effect of Previous Target Modality (F (2, 351) = 4.18, *p* = .014). Responses to targets following auditory targets were faster than visual (*p* < .001, d = 0.38; 95% CI [0.21, 0.53]) and tactile (*p* < .001, d = 0.23 95% CI [0.06, 0.40]). The difference between shifts from visual and tactile targets did not reach statistical significance (*p* = .251, *d* = 0.14 95% CI [-0.03, 0.31]).

There was a significant Current Target x Previous Target Modality interaction (F (4, 351) = 15.97, *p* < .001). To unpack this interaction, we compared RT on repeat and shift trials for each target modality. Comparisons are given in Table 3. There was an MSE for each target modality whereby RTs on repeat trials were faster than on shift trials, although the comparisons of visual and tactile repeat trials with previous trial auditory targets did not reach statistical significance.

**Table 3:** Comparison of reaction times (RTs) on shift and repeat trials (conditions called target – previous target; Bonferroni adjusted α = .008)

Visual - Visual vs	*p*	d 95% CI
Visual – Tactile	.002	0.35 [0.10, 0.56]
Visual – Auditory	.012	0.31 [0.06, 0.52]
**Tactile – Tactile vs.**		
Tactile – Visual	.004	0.39 [0.14, 0.59]
Tactile – Auditory	.020	0.26 [0.02 0.49]
**Auditory – Auditory vs**		
Auditory – Visual	< .001	0.81 [0.50, 0.93]
Auditory - Tactile	< .001	0.76 [0.47, 0.90]

**Fig. 2:**
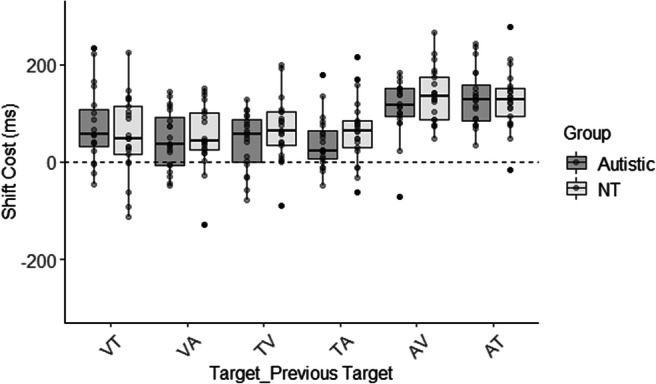
Shift costs for each target modality. The autistic group are shown in grey and the neurotypical group (NT) in white. Shift costs were calculated as the reaction time on shift trials – repeat trials

There was no main effect of Group (F (1, 351) = 0.02, *p* = .900), suggesting that RTs were comparable between the groups. Group did not interact with Current Target (F (2, 351) = 0.75, *p* = .473) nor Previous Target (F (2,351) = 0.75, *p* = .474), suggesting that RTs were similar in response to the different targets and when shifting. Critically, there was no three-way interaction (F (4,351) = 0.12, *p* = .975), suggesting that shift costs were similar for each target modality between the groups (see Fig. 2).

### Interim summary

MSEs with large effect sizes were observed for each target modality whereby RTs to visual, tactile and auditory targets were fastest on repeat trials in comparison with shift trials. This replicates previous studies that have reported exogenous MSEs for vision, touch and hearing (Miles et al., 2011; Spence et al., 2001; Tollner et al., 2009). We did not observe any differences between the groups in RT shift costs for any modality pairing using a design that addressed a number of methodological issues inherent in previous work. This is similar to previous studies that have used discrimination tasks to measure MSEs in autism (see Haigh et al., 2016; Murphy et al., 2014). However, our approach, in common with all previous work on this topic, does not deal with error rates or speed-accuracy trade-offs effectively. Responses would be expected to be faster *and* more accurate in shift conditions if the MSE is a consequence of a genuine perceptual effect (rather than, for instance, differences in response criterion for repeat and shift conditions; see Spence et al., 1997). However, analysis of shift costs in RT and error data separately cannot account for non-decision factors that can contaminate RT, such as delays in preparing the response. Moreover, RT and error data are not well correlated (Hedge et al., 2018), suggesting that they do not reflect the same underlying cognitive processes, as is widely assumed. Formal decision models incorporating RT and error data can address these issues, and are increasingly being applied to better understand cognitive processes in clinical groups (White et al., 2010). As such, we modelled participant RT and error data in an additional analysis in order to estimate drift diffusion model parameters (Ratcliff & McKoon, 2008; Voss et al., 2015) for trials involving a modality shift and a repeat, and compared parameters between the autistic and NT groups.

### Additional analysis: Drift diffusion model

The drift diffusion model is a framework for characterising the cognitive processes underlying a participant’s response (accuracy and RT) on a two-choice task. Selecting a response is described as a process of accumulation of evidence over time (Ratcliff & McKoon, 2008; Voss et al., 2013). Modelling the data in this way allows a number of parameters to be extracted from the participant’s response that have meaningful interpretations. *Drift rate* (*v*) is a measure of the quality of information extracted from the stimulus. A higher rate of drift indicates that the stimulus has been encoded more effectively and the quality of the information extracted is increased, meaning that the participant will make a faster, less error-prone response. This is therefore an index of the participant’s sensitivity to the stimulus. The *boundary separation* (*a*) is a measure of response conservativeness, where larger values represent a more conservative decision as more evidence must be accumulated before one of the boundaries is reached. *Non-decision time* (*T*_*0*_) is an estimate of the time taken for all non-decision-making aspects of the process, encompassing the encoding of the stimulus and the preparation of the response output

There are a number of advantages to using diffusion models when investigating cognitive processing in clinical groups (White et al., 2010). As described above, the model provides a theoretical framework for understanding the cognitive processes that underlie the participant’s response. Furthermore, in using RT on both correct and error trials, the entire response distribution is analysed. Analysis of diffusion model parameters can lead to a substantial increase in statistical power for between-group comparisons (Stafford et al., 2020), which is valuable when conducting experimental studies with clinical populations where sample sizes tend to be small. Recent work has used the diffusion model to understand autistic participants’ performance on orientation judgement tasks and has suggested that between-group differences relate to increased response conservativeness (increased boundary separation, *a*) in autistic participants rather than perceptual differences (differences in drift rate, *v*; Pirrone et al., 2017; Pirrone et al., 2018). Furthermore, a study of face recognition has shown that autistic children have reduced drift rates and boundary separation, suggesting that information was processed less efficiently and criterion setting was less conservative (Powell et al., 2019). Modelling responses in this way is emerging as a valuable approach to better understanding the cognitive mechanisms underlying differences in task performance in autism. Additionally, no previous work of the MSE has effectively combined RT and accuracy, and as such, this analysis can generate insight into the cognitive processes underlying the effect.

We anticipated that effortful shifting of attention towards the target in shift conditions should reduce the amount of information obtained from the stimulus leading to a reduction in drift rates compared with repeat conditions. If attentional weight shifting between the senses is less efficient in autism, then the autistic group would be expected to produce increased shift costs in the drift rate data (i.e., a greater difference in drift rates between repeat and shift conditions compared with the NT group). We anticipated that non-decision time would be increased in the shift conditions compared with the repeat conditions as more time would be required to encode the stimulus following the shift in attention between the senses. Shift costs in non-decision time were expected to be increased in the autistic group compared with NTs.

### Analysis

To give sufficient trial numbers to extract stable parameter estimates, responses to targets in different modalities were pooled to give responses on shift and repeat trials. A procedure for removing individual participant outliers similar to that described above, was used for RTs *across* correct and error responses in the shift and repeat conditions. Diffusion model parameters were estimated for each shift and response trial for each participant using the Kolmogrov-Smirnov procedure as implemented using fast-dm 30 (Voss et al., 2015). This is a robust and efficient method of parameter estimation for trial numbers > 100 (Voss et al., 2013). Three parameters were left free to vary in the fitting procedure: drift rate (*v*), boundary separation (α) and non-decision time (*t*_0_). Starting point was set at 0.5 and the intertrial variability of start point and drift rate were set to zero (as recommended by Lerche & Voss, 2016). As we were interested in the difference in model parameters between shift and repeat conditions, we did not analyse boundary separation here. The boundary separation is generally understood to be set at the beginning of the experiment, or adjusted before beginning a block of trials following some manipulation, such as instructions from the experimenter (Ratcliff & Smith, 2004).

**Fig. 3:**
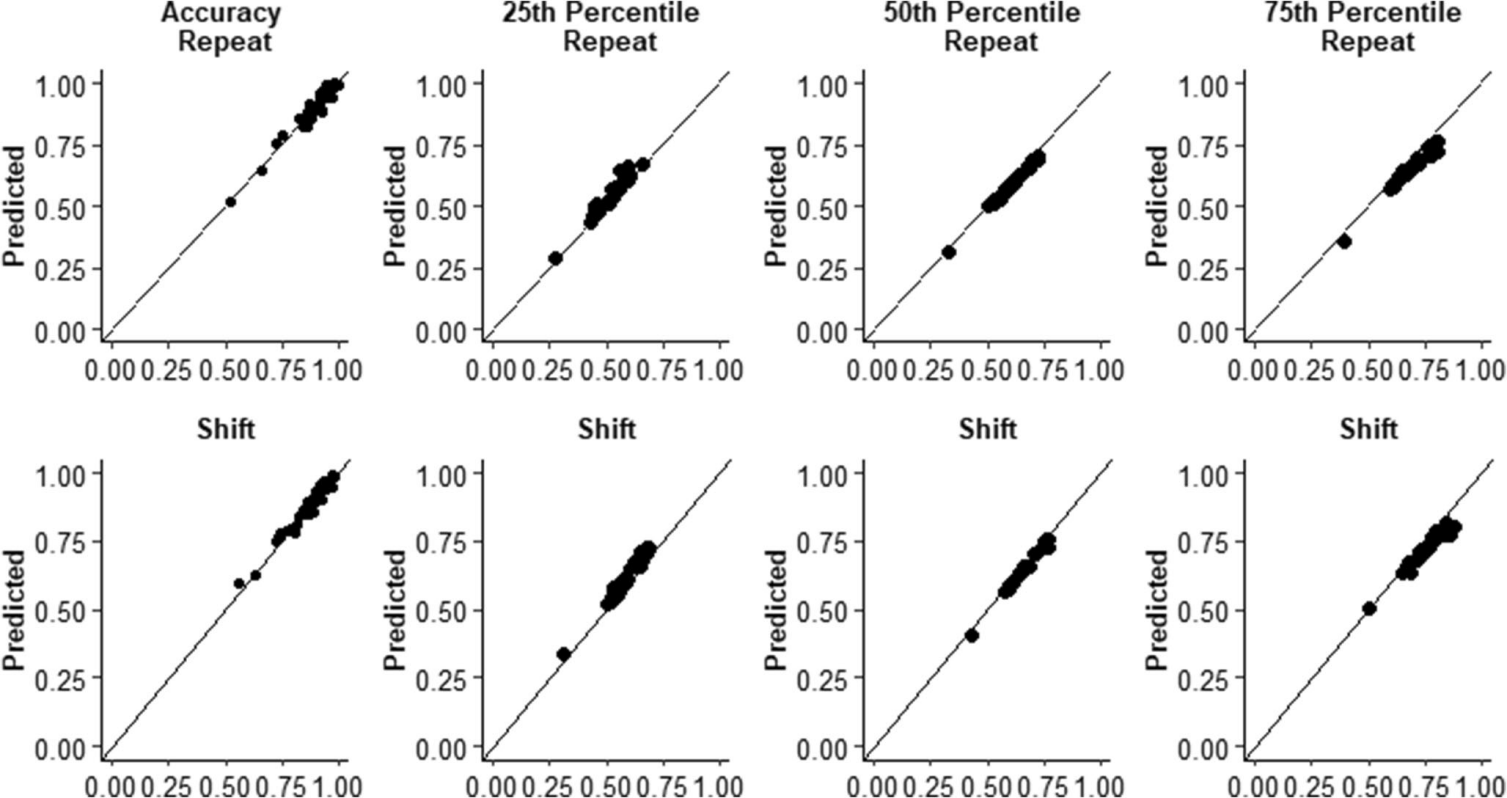
QQ plots displaying empirical and predictions of accuracy, and the 25th, 50th and 75th percentiles of the reaction-time distributions. Data points give individual participant empirical and predicted scores, points falling close to the x = y line indicate the model fits provided a good estimate of the empirical data

To assess model fits of the empirical data, 1,000 trials were sampled from the parameter estimates for each participant (using construct-samples in fast-dm-30; see Voss et al., 2015). Proportion accuracy, the 25th, 50th and 75th percentiles of the RT distribution were calculated for the predicted and empirical data and were plotted in QQ-Plots (see Fig. 3).

Drift rate and non-decision time were analysed using mixed ANOVA with Previous Target [Shift, Repeat] as the within- and Group [Autistic, NT] as the between-participant factor. The critical test of differences in shift costs between the groups was the interaction.

## Results

**Fig. 4:**
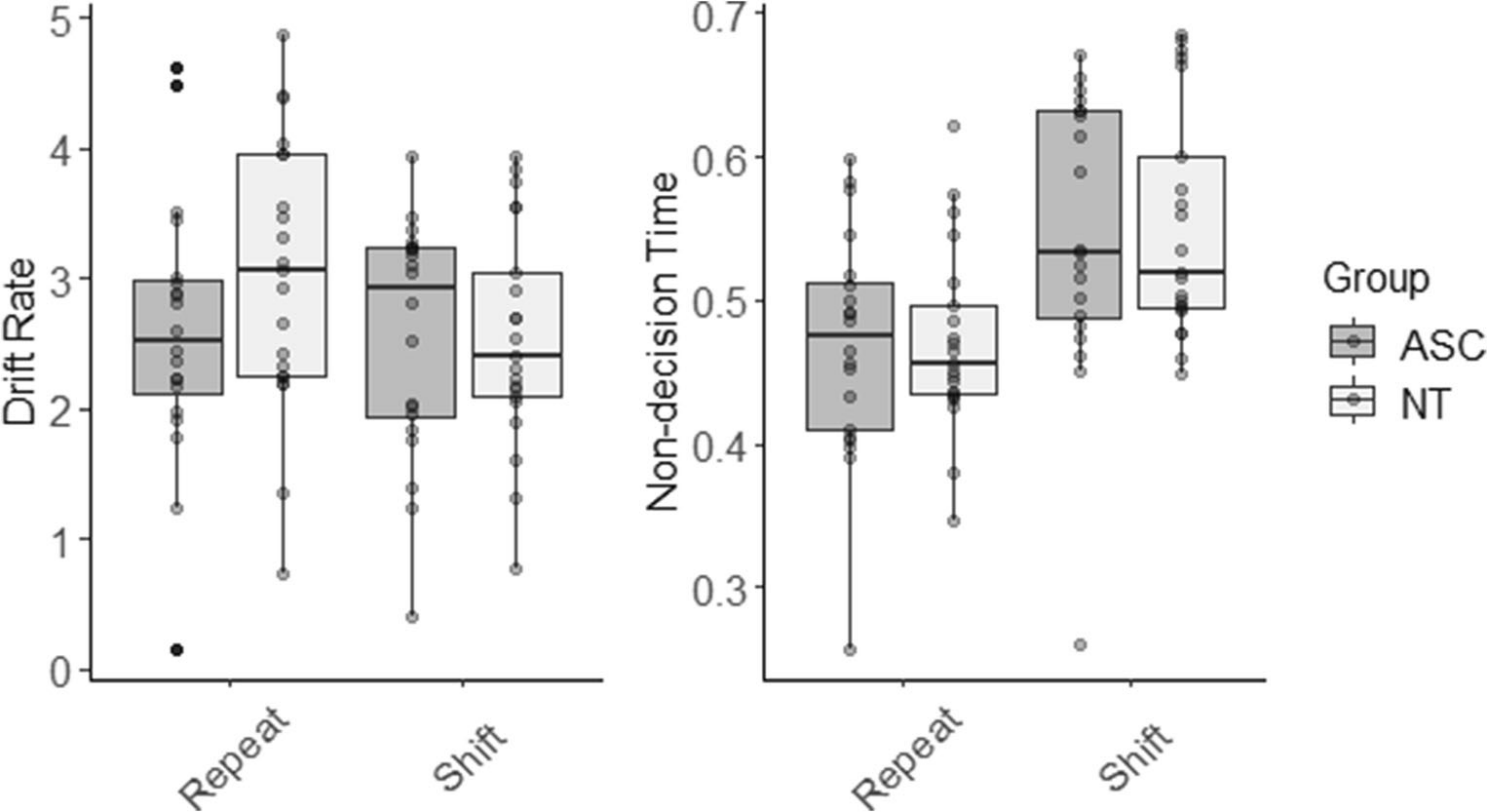
Boxplots displaying estimated diffusion model parameters (drift rate and non-decision time). The autistic group are shown in grey and the neurotypical (NT) group in white. For the NT group, drift rate was increased in repeat trials relative to shift, an MSE effect. This effect was not observed in the autistic group. Both groups showed an MSE in non-decision-time data with reduced non-decision time on repeat trials relative to shift

Estimated model parameters are displayed in Fig. 4. Drift rates were increased and non-decision time reduced on repeat trials in comparison with shift trials. Interestingly, there was a *reduced* shift cost for the autistic group in the drift rate data.

### Drift rate

There was an MSE in estimates of drift rates, such that values of v were reduced on shift trials (2.54 ± 0.87) in comparison with repeat trials (2.81 ± 1.04). This was confirmed using a mixed ANOVA, which revealed a main effect of condition (F (1,39) = 7.72, *p* = .008), indicating that drift rates were increased on repeat trials. There was no main effect of Group (F (1,39) = 0.55, *p* = .462), indicating that overall drift rate was comparable between the groups. The interaction between Condition and Group (F (1, 39) = 5.90, *p* = .020) was statistically significant. Shift costs were calculated (drift rate for shift trials – repeat trials; note a more negative value indicates a larger shift cost) and were larger in the NT group (-0.45 ± 0.59) compared with the autistic group (-0.06 ± 0.45) (t (38) = 2.43, *p* = .020, 0.44 95%CI [0.07, 0.81], d = 0.76; 95%CI [0.10, 1.41]). Finally, two one-sample t-tests (Bonferroni-adjusted α = .025) comparing shift costs for each group with 0 revealed that shift costs were statistically significantly different from zero in the NT group (t(20) = 3.71, *p* = .001, -0.47 95% CI [-0.74, -0.21], d = 0.81, 95% CI [-0.14, 1.76]), but not in the autistic group (t (19) = 0.24, *p* = .809, -0.03 [-0.30, 0.24], d = 0.05 95% CI [-0.99, 0.88])

### Non-decision time

There was an MSE in estimates of non-decision time, such that values of T_0_ were increased for shift trials (.55 ± .09) compared with repeat trials (.47 ± .08). This was confirmed using a mixed ANOVA, which revealed a main effect of condition (F (1,39) = 142.01, *p* < .001). There was no main effect of Group (F (1,39) = 0.03, *p* = .875), nor Group x Condition interaction (F (1,39) = 0.05, *p* = .971).

## Discussion

In the present study we investigated exogenous shifts of attention between visual, tactile and auditory targets in autistic and NT participants. RT data revealed a clear modality-shift effect for visual, tactile and auditory targets, whereby responses were fastest on repeat trials in comparison with shift trials. This effect was largest for auditory targets where RTs on repeat trials were markedly reduced. Critically, RTs and shift costs were comparable between the groups.

The considerable performance benefit observed for auditory repeat trials is a novel finding that recalls early work suggesting that auditory warning signals are particularly effective in facilitating responses to subsequent targets (Posner et al., 1976). The previous study measuring MSEs between visual, tactile and auditory targets observed no difference in shift costs between the modalities on a spatial discrimination task (Spence et al., 2001). One consideration relates to modality appropriateness for the given task (Lukas et al., 2014). In the present study, the participant was required to make a judgement where timing rather than location was relevant (*was the stimulus pulsed or continuous?)*, precision is typically increased when making temporal judgements using auditory information (Hartcher-O’Brien et al., 2014; Jones et al., 2009; Rammsayer, 2014). In the context of the modality-weighting account of the MSE (Tollner et al., 2009), it may be that modality appropriateness confers an additional enhancement in signal salience and up-weights attention priority towards subsequent signals, leading to the large RT benefit observed on auditory repeat trials. Alternatively, the boost in signal salience may instead be related to the relative perceived intensity of the crossmodal stimuli. On a repeat trial, the participant processes two stimuli of identical intensity, whereas a shift trial will likely also involve a change in perceived intensity. Where a target modality (i.e., auditory in the present study) is perceived as relatively more intense in comparison to other modalities, this could serve as an alerting signal for the subsequent target (Nissen, 1977). Further work could elaborate on the processes that modulate modality weight shifting by manipulating task requirements and the impact of the choice of the stimuli.

In an additional analysis, we examined participant responses using drift diffusion modelling, which combines RT and accuracy data. As anticipated, NT participants produced MSEs in both drift rate and non-decision-time data. According to the diffusion model framework (Ratcliff & McKoon, 2008; White et al., 2010), the increased drift rate following targets in the same modality reflects an increased rate of evidence accumulation, meaning the quality of information extracted from the stimulus was superior on repeat trials in comparison with shifts. Additionally, the reduced non-decision time suggests that the time taken to encode the stimulus and prepare the response was reduced on repeat trials in comparison with shift trials. This is highly consistent with the modality weight-shifting account of the MSE whereby up-weighting attentional allocation towards a modality was proposed to lead to an “enhanced coding of target signals within the weighted modality and/or enhanced transmission of modality specific target information” (Tollner et al., 2009). Interestingly, and in contrast to our hypothesis, shift costs in the drift rate data were *reduced* in the autistic group in comparison to NT controls, while non-decision times were comparable. This suggests that, unlike NTs, the quality of information extracted on repeat and shift trials was similar for autistic participants. As the autistic group showed similar shift costs in the non-decision-time data to controls, the time taken to encode the stimulus and prepare a response in either condition was likely to be similar. It may be sensory modality has less influence on attentional networks for autistic participants, meaning that within- and between-modality information is processed in a similar way. Relatedly, we previously observed that while NTs showed a benefit of inhibiting crossmodal distractors in comparison to within-modality distractors, autistic participants did not show this benefit (Poole et al., 2018). There are limited studies investigating crossmodal attention in autism, and it would be of value to probe the mechanisms whereby information within and between the senses is selected and inhibited, and whether differences are related to everyday sensory reactivity.

In the present study, we applied a principled methodological approach to measure MSEs in autistic and NT adults. When incorporating error rates into the analysis, we observed that shift costs were reduced in comparison to NTs. This effect was not apparent when considering RT data alone and contrasts with findings from the existing work. This highlights the advantage of using decision models (such as drift diffusion) to better characterise cognition in autism (as has been noted elsewhere; Pirrone et al., 2017, 2018; Powell et al., 2019). These models provide a theoretical framework of the cognitive processes involved in the participant’s response, integrating RT and accuracy data in a principled way, and increase power to observe between groups effects.

Although this study has provided a number of novel insights into the MSE, it is worth highlighting some limitations. Firstly, although the sample size for this study is typical of research investigating the MSE (see Table 1), statistical power to detect small to medium between-group effect sizes is low and this could account for the absence of an effect in the RT data. As recruiting participants from heterogenous populations such as autism (and appropriately matched control groups) is challenging, the use of diffusion modelling, which increases statistical power, would be recommended in follow-up studies of the MSE. Second, data from a small number of participants was discarded due to large numbers of miss trials. This may have been a consequence of the presentation of a rapid stream of crossmodal stimuli and/or recording rapid responses using a foot pedal, which some participants found difficult to co-ordinate. The foot pedal was used here as the dominant hand was occupied with the tactor, but future work could explore alternative methods of recording participant responses. The approach outlined in the current study would also be useful in unpacking the nature of the MSE in other neurodivergent conditions in which the MSE may be affected. To our knowledge, no previous work in participants with schizophrenia or dyslexia have utilised three target modalities, which has the advantage of allowing the efficiency of shifts to and from vision, touch and audition to be measured.

The present study comprised a thorough investigation of the exogenous shifting of attention between vision, touch and audition in autistic and NT participants. MSEs were observed for each target modality and were largest in response to auditory targets. Critically, there was no difference in RTs or shift costs between the groups. However, additional analysis using diffusion modelling revealed shift costs were reduced in the autistic participants’ drift rates, suggesting that the efficiency of processing ipsimodal and crossmodal stimuli was similar. This finding suggests there may be important differences in how autistic people attend to sensory modalities.
